# Perceived stress and reference ranges of hair cortisol in healthy adolescents

**DOI:** 10.1371/journal.pone.0214856

**Published:** 2019-04-04

**Authors:** Vicente Prado-Gascó, Usue de la Barrera, Sandra Sancho-Castillo, José Enrique de la Rubia-Ortí, Inmaculada Montoya-Castilla

**Affiliations:** 1 Department of Social Psychology, University of Valencia, Valencia, Spain; 2 Department of Personality, Assessment and Psychological Treatment, University of Valencia, Valencia, Spain; 3 Department of Basic Medical Sciences, Catholic University of Valencia, Valencia, Spain; University College London, UNITED KINGDOM

## Abstract

**Background:**

Chronic stress during adolescence has usually been evaluated through subjective measures, leaving aside objective measures such as hair cortisol concentrations. Therefore, the aim of this study was to provide reference ranges for hair cortisol concentrations by sex and age and to study the relationship between subjective and objective measures of stress and temporal stability.

**Methods:**

The participants were 170 adolescents aged between 12 and 14 years (*mean* = 12.78 years; *standard deviation* = 0.71 years; 52.40% girls) who completed the Perceived Stress Scale 4 and had their hair sampled.

**Results:**

The results revealed hair cortisol concentrations ranging from 0.07 pg/mg to 9.54 pg/mg. Subjective and objective measures of stress were not related, nor was there intraindividual stability of the hair cortisol concentrations. Girls had higher hair cortisol concentrations, and there were no age differences.

**Conclusions:**

This research provides cortisol reference values for adolescents that will allow the early detection of chronic stress. Such detection methods make it possible to prevent problems arising from stress because we can act more quickly and the treatments will be more effective. The study suggests that there is no relationship between perceived and objective stress; while perceived stress remained stable, the levels of hair cortisol were increased at 6 months. Despite the interesting findings of the study, there are some limitations: the sample was not obtained through probabilistic sampling, the age range was narrow, and some demographic, anthropomorphic and clinical factors are missing, which make the generalization of results difficult.

## Introduction

Stress is a common and frequent experience for most adolescents worldwide [1–4], and it is becoming a public health problem affecting adults and children alike [5]. Although stress is not necessarily associated with negative aspects, high or continuous levels of stress or scarce coping resources can make it a trigger for physical and mental health problems [6] that affect the quality of life [7, 8]. In this sense, stress appears to be associated with the onset of emotional problems and mental disorders, such as depression [8, 9], anxiety [10] and posttraumatic stress disorder [11]. This relationship between stress and health is particularly worrisome during adolescence, a period of great vulnerability [12, 13]. At this stage, academic, social, and family pressures along with rapid changes in the environment and in adolescents themselves lead to high levels of stress [14–16].

As a result, in recent years, there has been a growing interest in analyzing stress levels during adolescence. Subjective measures such as questionnaires have usually been used [6, 17–19]. However, although children and adults provide good information about their internal experiences [20], such measures can be strongly influenced by personal or subjective aspects; therefore, it is necessary to include objective measures of stress to determine the physiological response. Of the existing indicators, cortisol is considered the most reliable biomarker for measuring physiological stress levels [21]. Cortisol may be considered the primary glucocorticoid in humans [22], and it plays a critical role in the stress response and the protection of the body and brain [23]. The mechanism underlying the relationship between stress and cortisol is as follows: the hypothalamic-pituitary-adrenal axis (HPA) regulates various body processes and interactions, including reactions to psychological stress [24–26]. The release of glucocorticoids, such as cortisol, functions as a negative feedback regulator of the HPA axis.

To date, most studies have analyzed cortisol responses using saliva, urine, or serum samples [27–30]. However, these methods reflect acute or short-term hormone levels [31], which often have daily fluctuations. In contrast, the analysis of hair cortisol concentrations (HCCs) provides a reliable and valid long-term index of cortisol secretion [32–36]. This makes HCC analysis a very attractive measure for determining chronic stress [37, 38], especially in the youth population, for which rapid, noninvasive sampling procedures are essential.

Although both subjective and objective measures are used to determine chronic stress levels in adolescents, the relationship between these types of measures is controversial [39]. In some studies, HCC correlates positively and significantly with perceived stress measures [40–42]. However, other studies do not find such a relationship [43–46] or find a negative relationship [47]. It therefore seems necessary to deepen the investigation into this relationship.

Despite the importance of research that uses both subjective and objective measures, such studies are scarce in healthy adolescents [46]. Most research on HCCs has been conducted in the adult population [35], and there are few studies in children and adolescents [48]. In this regard, Stalder et al. [46] performed a meta-analysis of research evaluating HCCs in healthy people, and only 13 of 124 subsamples included subjects under 18 years of age. In comparison, a systematic review by Gray et al. [49] included 36 studies carried out with minors, but only 10 of them included adolescents. Of the 10 studies, four of the investigations included adolescents with diseases such as asthma [50], anorexia nervosa [51], adrenal insufficiency [52] and chronic psychological stress [53]; one investigation focused on adolescents living in socioeconomically disadvantaged neighborhoods [54], and another focused on children and adolescents experiencing maltreatment [55]. Therefore, only 4 of these 10 studies focused exclusively on healthy adolescents. It should be noted that only one of the studies offered HCC reference ranges [28]. However, these ranges were not differentiated by sex, the temporal stability of HCC was not evaluated, and the sample included only 128 subjects. In relation to the stability of HCC measurements in adolescents, although research with 17- to 21-year-olds indicates that there may be some intraindividual stability [56], there is limited research on adolescents. In this context, it is essential to carry out research to fill this gap in the literature.

According to the literature, stress levels in adolescents, whether subjective or physiological, seem to be affected by demographic variables, such as sex and age [57]. On the one hand, girls have higher cortisol levels than boys [58] and report more perceived stress [17, 59]. However, in terms of cortisol levels, some studies have found that boys show higher levels than girls [35, 60], while others have found no difference between the two [28, 57, 61, 62]. Controversy regarding age also seems to exist. Some studies suggest that there is no association between age and HCC [35, 50, 51, 60, 63–66], while others have found a positive relationship [28, 55]. This discrepancy highlights the need for studies to clarify the possible influence of age on HCC [49] and sex on stress levels [46, 62]. The abovementioned issues highlight the importance of the study presented here.

First, given the high impact of stress on health, especially among adolescents, and the paucity of studies that examine HCCs in young people, research is needed to determine HCCs in healthy adolescents. Second, considering the controversial relationship between perceived stress and HCCs and the lack of consistent data on this relationship [39], especially during a developmental period of great vulnerability (namely, adolescence) and in a population that is rarely considered (namely, healthy people), it is critical to analyze this relationship in this type of population. Third, because of the limited studies on the temporal stability of HCC measurement in healthy adolescents, it is necessary to analyze possible fluctuations in cortisol level at two time points (6 months apart). Finally, there does not seem to be any agreement in the literature on the role of sex and age in stress levels, especially with regard to objective measures; thus, further studies are needed to analyze the impact of sex and age by considering both objective and subjective measures.

In this context, the objectives of the present investigation were to identify HCC reference ranges in healthy adolescents, examine the relationship between perceived stress and cortisol levels, determine the temporal stability of HCCs and perceived stress at 6 months and analyze the roles of sex and age in subjective and objective stress measures. The hypotheses were that subjective and objective measurements of stress will be positively related [40–42] and that the HCC will remain stable over 6 months. Regarding sex, there will be differences only in perceived stress levels, with girls scoring higher [17, 59]. With respect to age, adolescents between the ages of 12 and 14 are around Tanner stage IV [67, 68]; considering the literature to date on cortisol levels during this stage, there will be no differences in either perceived stress levels or HCCs [35, 63]. Few studies have analyzed the HCCs of healthy adolescents in samples of this size; therefore, verifying whether differences exist could be interesting.

## Materials and methods

All procedures performed in studies involving human participants were in accordance with the ethical standards of the institutional and/or national research committee and with the 1964 Helsinki declaration and its later amendments or comparable ethical standards. The consent was obtained from the government, schools, parents and the university’s ethics commission, before adolescents were included in the study. It was approved by ethics committee.

### Participants

The participants were 170 healthy adolescents aged between 12 and 14 years old (*M* = 12.78 years; *SD* = 0.71 years; 52.40% girls) from 4 public schools. They were selected from a convenience sample of 1500 adolescents from 4 public schools in the Valencian Community who had previously provided informed consent to participate.

### Procedure

This study is part of a larger investigation funded by the Ministry of Economy and Competitiveness ("Enhancing psychological well-being and school coexistence in adolescents through education in emotions: longitudinal study" /PSI2013-43943-R). The necessary consent was obtained from the government, the schools, and the university’s ethics commission, before the adolescents were included in the study. Parents provided written, informed consent for their child to participate in the present study.

There were two principal inclusion criteria. First, the participants had to certify the absence of any disease that would influence their glucocorticoid levels (especially Addison’s disease and Cushing’s disease), including mental and psychiatric illness (including anxiety, depression, obsessive-compulsive disorder and attention-deficit/ hyperactivity disorder). Second, they had to certify that they were not receiving any pharmacological treatment (administered by any method: oral, inhaled or topical) that could influence cortisol levels, including antipsychotics, ADHD medications and corticosteroids. There was one principal exclusion criteria: the adolescents could not have dyed hair.

A longitudinal descriptive study was conducted to test the cortisol levels in the adolescents. During January 2016, the first hair collection was carried out, and the adolescents completed the questionnaire and provided sociodemographic data. A second evaluation was carried out 6 months later following the same procedure. Both hair samples were collected from the same posterior region of the head.

### Assessment and measures

#### Perceived stress

The Perceived Stress Scale 4 (PSS-4) [69, 70] is a reduced version of the Perceived Stress Scale (PSS) [71]. It is a self-report scale made up of four items. Its brevity makes it especially suitable for adolescents as it facilitates very rapid administration. The questionnaire assesses the extent to which the subject assesses his or her life over the past month as unpredictable, uncontrollable and overloaded. The response format consists of a 4-point Likert scale (1 = *never*; 4 = *always*). A higher score indicates a higher presence of perceived stress. Previous research has shown that this scale is reliable and valid [70], which was also observed in this study.

#### Hair cortisol concentrations

A 2-cm lock of hair from each participant (25–40 mg, approximately 150 hair strands) was cut from the back of the head, as close to the scalp as possible. This area has been shown to have the most stable HCC [72]. To prepare the samples for the extraction of the cortisol in the most efficient way, they were cut finely, one by one, using fine scissors, until they were practically a powder, so that when they were placed in contact with the methanol, maximum extraction was achieved. The hair locks were taped to a paper form with the scalp end marked and stored in individual sets at room temperature. Then, the samples were combined with alcohol and agitated at medium intensity for 36 hours at 37.7 degrees. After this process, methanol was extracted from each sample (with a 1000-μl pipette) and transferred to an Eppendorf tube. The alcohol was evaporated by means of an evaporator associated with nitrogen flow to improve the process, and the samples were placed on a hot plate to promote further evaporation. Cortisol was quantified using the Salivary Cortisol ELISA SLV-2930 kit (DRG International Inc.) after the solubilization of the pellet obtained after the evaporation of the methanol with a phosphate-buffered saline (PBS).

### Statistical analysis

The statistical analysis was conducted using SPSS 23.0. First, the descriptive statistics and reference range were calculated considering the whole sample, grouped by sex and age. Second, following the recommendations of the literature [73], a logarithmic transformation of the HCC variables was carried out. The relationship between cortisol level and PSS was calculated by means of a Pearson correlation considering the whole sample, grouped by sex and age. Third, the stability of the cortisol levels and the perceived stress were analyzed by means of a t-test. Finally, t-tests were performed to analyze the differences in cortisol levels considering sex, and ANOVA was used to analyze the differences by age.

## Results

### Cortisol levels

Table 1 presents the descriptive statistics and reference ranges for cortisol levels for the whole sample and by sex and age. The cortisol levels were between 0.07 pg/mg and 9.54 pg/mg (*M* = 3.26; *SD* = 1.88) for the entire sample. When considering the levels grouped by sex, we observed that in the case of girls, the cortisol levels ranged from 0.20 to 9.54 (*M* = 4.00; *SD* = 1.88), while in boys, the values ranged from 0.07 to 7.85 (*M* = 2.44; *SD* = 1.52). With respect to age, the range of cortisol values for 12-year-olds was found to range from 0.20 to 9.54 (*M* = 3.01; *SD* = 1.98); for 13-year-olds, from 0.07 to 7.88 (*M* = 3.62; *SD* = 1.84); and for 14-year-olds, from 0.37 to 6.96 (*M* = 2.86; *SD* = 1.61).

**Table 1:** Descriptive statistics and HCC reference ranges (pg/mg) for the whole sample and by sex and age.

		Whole sample (*n* = 170)	Girls (*n* = 88)	Boys (*n* = 80)	12 years (*n* = 65)	13 years (*n* = 75)	14 years (*n* = 28)
Mean		3.26	4.00	2.44	3.01	3.62	2.86
Standard deviation		1.87	1.88	1.52	1.98	1.84	1.61
Minimum		0.07	0.20	0.07	0.20	0.07	0.37
Maximum		9.54	9.54	7.85	9.54	7.88	6.96
Percentile	10	0.92	1.58	0.70	0.59	1.26	0.88
	20	1.60	2.57	1.11	1.23	1.72	1.58
	25	1.80	2.74	1.47	1.55	2.03	1.67
	30	2.13	2.98	1.55	1.84	2.47	2.02
	40	2.62	3.34	1.82	2.46	2.98	2.34
	50	3.02	3.80	2.18	2.88	3.77	2.64
	60	3.54	4.30	2.60	3.25	4.26	2.83
	70	4.02	4.82	2.99	3.67	4.64	3.33
	75	4.36	5.35	3.35	3.85	4.80	3.66
	80	4.70	5.69	3.71	4.02	5.05	3.82
	90	5.85	6.74	4.37	6.10	5.92	5.47

### Relation between perceived stress (PSS) and cortisol levels

The relationship between perceived stress and cortisol levels was analyzed with a Pearson correlation for the whole sample and by sex and age. According to the results at time 1 (*r* = .07, *p* = .381) and time 2 (*r* = -.11, *p* = .411), no correlation was found for the whole sample. When the role of sex and age in that relationship was examined, no relationship was observed for girls (*r* = .10, *p* = .353), boys (*r* = .07, *p* = .521), adolescents at 12 years old (*r* = .01, *p* = .961), adolescents at 13 years old (*r* = .20, *p* = .086) or adolescents at 14 years old (*r* = -.14, *p* = .477) at time one.

### Stability of cortisol levels at 6 months

Then, to analyze the stability of cortisol levels, new measurements were taken after six months, and a t-test was performed considering both moments for a subsample of 76 adolescents of the total sample of 170 (Table 2). The age and sex distributions of the participants included in the subsample were similar to those of the excluded participants. Considering the cortisol levels at moment 1, the values ranged between 0.22 and 7.88 (*M* = 4.08; *SD* = 1.79), while at moment 2, the values ranged between 1.91 and 9.27 (*M* = 4.88; *SD* = 1.70). According to the t-test results, there seemed to be stability in perceived stress after 6 months [*t*(51) = -1.30; *p* = .201; Cohen’s *d* = -0.22], but not in cortisol levels [*t*(75) = -3.46; *p* = .001; Cohen’s *d* = -0.45].

**Table 2:** Stability of HCCs at 6 months.

	Time 1		Time 2		*t*	*p*	Cohen’s *d* [95% CI]^a^
	*M*	*SD*	*M*	*SD*			
HCCs^b^	1.27	0.61	1.52	0.36	-3.46	0.001	-0.45 [-0.75, -0.14]
PSS^c^	8.17	2.29	8.65	2.10	-1.30	0.201	-0.22 [-0.64, 0.20]

^a^Size effect (confidence interval)

^b^Hair cortisol concentrations

^c^Perceived Stress Scale

### Differences in cortisol and perceived stress levels considering sex and age

Differences in hair cortisol levels and perceived stress levels were analyzed according to sex and age. Considering sex (Table 3), there were statistically significant differences in cortisol levels [*t*(166) = 5.02; *p* < .001; Cohen’s *d* = 0.91], with the girls showing higher levels than the boys (girls: *M* = 1.23; *SD* = 0.68; boys: *M* = 0.65; *SD* = 0.81). However, no such differences in perceived stress levels were observed [*t*(159) = -0.19; *p* = .848; Cohen’s *d* = -0.04]. Additionally, according to age (Table 4), there were no statistically significant differences in the levels of cortisol in hair [*F*(2) = 2.09; *p* = .126; η^2^ = .03] nor in the levels of perceived stress [*F*(2) = 0.21; *p* = .814; η^2^ = .00] among 12, 13 and 14-year-olds.

**Table 3:** Mean difference in physiological and perceived stress between girls and boys.

	Girls			Boys			*t*	*p*	Cohen’s *d* [95% CI]^a^
	*n*	*M*	*SD*	*n*	*M*	*SD*			
HCCs^b^	88	1.23	0.68	80	0.65	0.81	5.01	< .001	0.91 [0.66, 1.17]
PSS^c^	84	8.14	2.22	77	8.21	2.05	-0.19	.848	-0.04 [-0.36, 0.20]

^a^Size effect (confidence interval)

^b^Hair cortisol concentrations

^c^Perceived Stress Scale

**Table 4:** Mean difference in physiological and perceived stress according to age.

	12 years			13 years			14 years			*F*	*p*	η^2^^a^
	*n*	*M*	*SD*	*n*	*M*	*SD*	*n*	*M*	*SD*			
HCCs^b^	65	0.82	0.87	75	1.09	0.77	28	0.87	0.67	2.09	.126	0.03
PSS^c^	61	8.28	1.95	72	8.17	2.33	28	7.96	2.06	0.21	.814	0.00

^a^Eta square (size effect)

^b^Hair cortisol concentrations

^c^Perceived Stress Scale

## Discussion

As noted above, stress, although a common experience in adolescence [15], is a determinant of physical and mental health [7], especially in young people [6]. Therefore, the importance of this study is highlighted, given the gaps in the literature regarding the analysis of stress in healthy adolescents. There are few studies combining subjective and physiological measures, and there is no consensus on the relationship between the two types of measures. There is also controversy regarding the role of age and sex in stress levels, and there are no reference ranges for the HCC in healthy adolescents that account for the temporal stability of the measure.

The aims of the present study were to determine the levels of subjective stress and the HCCs in healthy adolescents, to offer HCC reference ranges, to analyze the relationship between perceived and physiological stress, to study the differences in stress levels according to sex and age and to determine the temporal stability of HCCs during adolescence.

The results suggest that the levels of cortisol in adolescents range from 0.07 pg/mg to 9.54 pg/mg. HCC reference ranges are also offered by sex and age. Most research on HCCs has been conducted in adults [35], and there are few studies in young people [48]. This research provides HCC reference ranges for adolescents, which will allow the interpretation of this measure in healthy young people.

Regarding the relationship between perceived stress and cortisol levels, the results obtained showed no association between the two measures for either the whole sample by sex and age. These results are not in line with the starting hypothesis but are consistent with some previous studies [43–46]. Stress is a complex phenomenon that manifests at different levels, including the physiological, cognitive, behavioral and emotional. The HCC captures only one aspect of endocrine activity and cannot fully reflect the functioning of the HPA axis [46]. The PSS focuses on the subjective perception of stress, which is the most cognitive aspect. The absence of an association between the two measures may be explained by the fact that they assess responses other than stress that do not necessarily have to occur at the same time. It is possible that some adolescents, when faced with stress, may have more physiological manifestations and others may have more cognitive manifestations, but not both simultaneously. Therefore, young people who exhibit more physiological stress may or may not have more subjective stress, and vice versa. Another reason may be that PSS scores and cortisol levels are parameters that depend on very different factors that may influence each parameter differently [43]. These results may clarify the relationship between the two constructs, which has hardly been studied in the literature.

The hypothesis regarding the temporal stability of stress was that it would remain stable for 6 months, but the results only partially confirmed this hypothesis. The results suggest that perceived stress remained stable, but HCC values were higher after 6 months. Since there is no relationship between perceived stress and physiological stress [43–46], differences in stability could be found depending on the type of measure used.

The differences in HCC at the two time points could be due to different explanations. First, the literature suggests that cortisol secretion fluctuates depending on the time of year [27]; in the present research, the first stress measurement was performed in January, and the second was performed in June.

Second, during the T2 assessment, the adolescents were in the midst of their examination period, a few days before the end of the academic year; during this period, they usually have a more negative affect [74]. In our study, the adolescents experienced more objective stress, but no differences were found in perceived stress, perhaps because the adolescents had difficulty perceiving stress, even when they were suffering from it. In this sense, the adolescents may have become accustomed to that stressful period; although they may not have been able to detect stress subjectively, their bodies detected it. Therefore, their cortisol levels were higher during that period [74] despite an absence of fluctuations in perceived stress, as observed in our research.

Finally, another explanation might be the adolescents’ assessments of the stressful situation and their own resources for coping with those situations, as is pointed out in the Lazarus model [75]. From this perspective, a person may experience more objective stress (HCC), but his or her subjective perception may be modulated by those other factors.

Nevertheless, the literature suggests that the relationship between objective and subjective stress, as well as the temporal stability of stress levels according to different measures, are not clear. Therefore, these results are especially interesting and novel given the scarcity of studies measuring the stability of HCC and perceived stress in healthy adolescents. Nonetheless, it is necessary to continue to deepen the comparison of both types of measures at different times both with other samples and in other contexts.

Finally, considering the differences according to sex and age, we observed that in the case of the girls, the cortisol levels ranged from 0.20 pg/mg to 9.54 pg/mg, while for the boys, the values ranged from 0.07 pg/mg to 7.85 pg/mg. The cortisol values of the 12-year-olds ranged from 0.20 pg/mg to 9.54 pg/mg; in the13-year-olds, they ranged from 0.07 pg/mg to 7.88 pg/mg; and in the 14-year-olds, they ranged from 0.37 pg/mg to 6.96 pg/mg. Based on these results, the third hypothesis was not supported by the data, but the fourth hypothesis was. There were significant differences in cortisol levels by sex, and the girls had higher values. These differences were not observed for subjective stress levels. Despite not confirming the third hypothesis, these HCC results are in line with previous research [17, 58, 59] and suggest that girls experience higher levels of chronic stress than boys. As noted above, little is known about the impact of sex on stress levels in healthy adolescents, especially with regard to the HCC. The present research helps to fill this gap in the literature. The results seem to support the final hypothesis of the study: the levels of perceived stress and physiological stress do not seem to vary according to age in adolescents aged 12 to 14 years. These results are in line with previous studies in which there was no link between age and stress [35, 63, 64, 66]. This research provides knowledge that allows us to extend and consolidate the limited results observed in the literature.

Despite the interesting findings of the study presented here, it is not exempt from limitations. First, the sample was not obtained through probabilistic sampling, and information was only collected from adolescents in the Valencian Community, which makes it difficult to generalize the results. It would be advisable to extend the sample to other Spanish populations. Second, the presence of exams and the time of year at which the data were obtained may have influenced the results of this research. Third, some demographic, anthropomorphic and clinical factors that may have helped to better characterize the sample are missing; these factors include IQ, BMI, substance use, hospitalizations, and stress during the early years of life. Fourth, the adolescents were in the pubertal state (Tanner stage IV) and their age range was narrow [67, 68], so the results on the effect of age on study variables should be taken with caution. Five, the PSS-4 assessment only asks questions about life during the past month. Hence, the hair correlating to this time period was the proximal 1 cm; however, we collected 2 cm of hair, which would correspond with the cortisol levels over the previous two months. However, the PSS-4 is an adapted and validated measure, and modifications of its wording by changing the temporal period of consideration are not recommended. Finally, no account was taken of whether the participants had acute inflammatory conditions. To avoid this limitation in future research, participants should certify the absence of acute inflammatory conditions.

Despite the limitations, this research is especially useful for the prevention and promotion of adolescent health as it advances the measurement and understanding of stress in healthy populations during adolescence, a complex and stressful period. The research fills some of the gaps in the literature and improves the understanding of stress by offering, among other aspects, reference ranges for HCC differentiated by sex and age, which do not exist in the current literature. In this way, as a result of the improved detection of stress levels and the existence of comparative parameters, it will be possible to prevent the emergence of emotional problems associated with stress during adolescence [8–10], which will result in an improvement in the physical and mental health of young people and higher levels of well-being.

Among the contributions of the present study are its provision of HCC reference ranges for healthy adolescents, its use of a considerably larger sample than in most HCC studies to date, its focus on an understudied population, and its analysis of the temporal stability of HCC over a period of 6 months—an aspect that has been minimally evaluated to date in healthy adolescents. The present study also combines objective and subjective measures to assess stress and examines the relationship between the two types of measures. Finally, it explores the impact of sex and age on the stress levels of adolescents, an aspect that has rarely been analyzed in the scientific literature.

## Supporting information

S1 Dataset BBDD_T1_Cortisol. (DAT)

S2 Dataset BBDD_T1T2_Cortisol. (DAT)

## References

[1] Brown BB, Larson RW, Saraswathi TS, editors. The world’s youth. Adolescence in eight regions around the globe. Cambridge, UK: Cambridge University Press; 2002.

[2] Gelhaar T, Seiffge-Krenke I, Borge A, Cicognani E, Cunha M, Loncaric D, et al Adolescent coping with everyday stressors: A seven-nation study with youth from central, eastern, southern and northern Europe. Eur J Dev Psychol. 2007; 4:129–56. 10.1080/17405620600831564

[3] Persike M, Seiffge-Krenke I. Competence in Coping with Stress in Adolescents from Three Regions of the World. J Youth Adolesc. 2012; 41:863–79. 10.1007/s10964-011-9719-6 21983844

[4] Cameron CA, McKay S, Wynne-Edwards K, Wright JM, Weinberg J. Cortisol Stress Response Variability in Early Adolescence: Attachment, Affect and Sex. J Youth Adolesc. 2017; 46:104–20. 10.1007/s10964-016-0548-5 27468997 PMC5518686

[5] Anderson NB, Nordal KC, Breckler SJ, Ballard D, Bufka L, Bossolo L, et al Stress in America. Monitor on Psychology [internet]. 2011; 41(1): 1–68. Available from: http://www.apa.org/monitor/2011/01/stressed-america.aspx

[6] Moksnes UK, Haugan G. Stressor experience negatively affects life satisfaction in adolescents: the positive role of sense of coherence. Qual Life Res. 2015; 24:2473–81. 10.1007/s11136-015-0977-8 25833012

[7] Burkhart ML, Horn-Mallers M, Bono KE. Daily Reports of Stress, Mood, and Physical Health in Middle Childhood. J Child Fam Stud. 2017; 26:1345–55. 10.1007/s10826-017-0665-0

[8] Shapero BG, McClung G, Bangasser DA, Abramson LY, Alloy LB. Interaction of Biological Stress Recovery and Cognitive Vulnerability for Depression in Adolescence. J Youth Adolesc. 2017; 46:91–103. 10.1007/s10964-016-0451-0 26923989 PMC5003774

[9] Steudte-Schmiedgen S, Wichmann S, Stalder T, Hilbert K, Muehlhan M, Lueken U, et al Hair cortisol concentrations and cortisol stress reactivity in generalized anxiety disorder, major depression and their comorbidity. J Psychiat Res. 2017; 84:184–90. 10.1016/j.jpsychires.2016.09.024 27744230

[10] McEwen BS, Bowles NP, Gray JD, Hill MN, Hunter RG, Karatsoreos IN, Nasca C. Mechanisms of stress in the brain. Nat Neurosci. 2015; 18(10):1353–63. 10.1038/nn.4086 26404710 PMC4933289

[11] Steudte S, Kirschbaum C, Gao W, Alexander N, Schönfeld S, Hoyer J, et al Hair Cortisol as a Biomarker of Traumatization in Healthy Individuals and Posttraumatic Stress Disorder Patients. Biol Psychiat. 2013; 74:639–46. 10.1016/j.biopsych.2013.03.011 23623187

[12] Romeo RD. Adolescence: A central event in shaping stress reactivity. Dev Psychobiol. 2010; 52(3):244–53. 10.1002/dev.20437 20175102

[13] Duarte JO, Cruz FC, Leão RM, Planeta CS, Crestani CC. Stress Vulnerability During Adolescence: Comparison of Chronic Stressors in Adolescent and Adult Rats. Psychosom Med. 2015; 77:186–99. 10.1097/PSY.0000000000000141 25659080

[14] Moksnes UK, Byrne DG, Mazanov J, Espnes GA. Adolescent stress: Evaluation of the factor structure of the Adolescent Stress Questionnaire. Scand J Psychol. 2010; 51, 203–9. 10.1111/j.1467-9450.2009.00803.x 20149144

[15] Bluth K, Roberson PNE, Gaylord SA, Faurot KR, Grewen KM, Arzon S, et al Does Self-Compassion Protect Adolescents from Stress? J Child Fam Stud. 2016; 25:1098–109. 10.1007/s10826-015-0307-3 26997856 PMC4793986

[16] Debnam KJ, Milam AJ, Mullen MM, Lacey K, Bradshaw CP. The Moderating Role of Spirituality in the Association between Stress and Substance Use among Adolescents: Differences by Gender. J Youth Adolesc. 2017; 47(4):818–28. 10.1007/s10964-017-0687-3 28493184

[17] Low NCP, Dugas E, O’Loughlin E, Rodriguez D, Contreras G, Chaiton M, O’Loughlin J. Common stressful life events and difficulties are associated with mental health symptoms and substance use in young adolescents. BMC Psychiatry. 2012; 12:116 10.1186/1471-244X-12-116 22900789 PMC3466152

[18] Zhang B, Yan X, Zhao F, Yuan F. The Relationship between Perceived Stress and Adolescent Depression: The Roles of Social Support and Gender. Soc Indic Res, 2015; 123:501–18. 10.1007/s11205-014-0739-y

[19] Burger K, Samuel R. The Role of Perceived Stress and Self-Efficacy in Young People’s Life Satisfaction: A Longitudinal Study. J Youth Adolesc. 2017; 46:78–90. 10.1007/s10964-016-0608-x 27812840

[20] Lundqvist C, Rugland E, Clench-Aas J, Bartonova A, Hofoss D. Children are reliable reporters of common symptoms: Results from a self-reported symptom diary for primary school children. Acta Paediat. 2010; 99(7):1054–59. 10.1111/j.1651-2227.2010.01727.x 20175756

[21] Chen N, Deater-Deckard K, Bell MA. The role of temperament by family environment interactions in child maladjustment. J Abnorm Child Psychol. 2014; 42(8):1251–62. 10.1007/s10802-014-9872-y 24691836 PMC4185014

[22] King SL, Hegadoren KM. Stress hormones: how do they measure up? Biol Res Nurs. 2002; 4(2):92–103. 10.1177/1099800402238334 12408215

[23] Salovey P, Stroud LR, Woolery A, Epel ES. Perceived emotional intelligence, stress reactivity, and symptom reports: Further explorations using the trait meta-mood scale. Psychol Health. 2002; 17(5):611–27. 10.1080/08870440290025812

[24] Herman JP, Cullinan WE. Neurocircuitry of stress: central control of the hypothalamo–pituitary–adrenocortical axis. Trends Neurosci. 1997; 20(2):78–84. 9023876 10.1016/s0166-2236(96)10069-2

[25] Kirschbaum C, Hellhammer DH. Salivary cortisol 2nd ed In: Fink G, editor. Encyclopedia of stress. Oxford: Academic Press; 2007 p. 405–9.

[26] Hellhammer DH, Wüst S, Kudielka BM. Salivary cortisol as a biomarker in stress research. Psychoneuroendocrinology. 2009; 34:163–71. 10.1016/j.psyneuen.2008.10.026 19095358

[27] Persson R, Garde AH, Hansen AM, Osterberg K, Larsson B, Orbaek P, Karlson B. Seasonal variation in human salivary cortisol concentration. Chronobiol Int. 2008; 25(6):923–37. 10.1080/07420520802553648 19005896

[28] Noppe G, van Rossum EF, Koper JW, Manenschijn L, Bruining GJ, de Rijke YB, et al Validation and Reference Ranges of Hair Cortisol Measurement in Healthy Children. Horm Res Paediatr. 2014; 82:97–102. 10.1159/000362519 25115629

[29] Geoffroy M-C, Hertzman C, Li L, Power C. Prospective Association of Morning Salivary Cortisol with Depressive Symptoms in Mid-Life: A Life-Course Study. PLoS ONE. 2013; 8(11): e77603 10.1371/journal.pone.0077603 24265676 PMC3827055

[30] Puig-Perez S, Pulopulos MM, Hidalgo V, Salvador A. Being an optimist or a pessimist and its relationship with morning cortisol release and past life review in healthy older people. Psychol Health. 2018; 33(6):783–99. 10.1080/08870446.2017.1408807 29166781

[31] Pruessner J, Kirschbaum C, Meinlschmid G, Hellhammer DH. Two formulas for computation of the area under the curve represent measures of total hormone concentration versus time-dependent change. Psychoneuroendocrinology. 2003; 28(7):916–91. 10.1016/S0306-4530(02)00108-7 12892658

[32] Gow R, Thomson S, Rieder M, Van Uum S, Koren G. An assessment of cortisol analysis in hair and its clinical applications. Forensic Sci Int. 2010; 196(1–3): 32–7. 10.1016/j.forsciint.2009.12.040 20096513

[33] Stalder T, Kirschbaum C. Analysis of cortisol in hair-state of the art and future directions. Brain Behav Immun. 2012; 26(7): 1019–29. 10.1016/j.bbi.2012.02.002 22366690

[34] Vanaelst B, Huybrechts I, Bammann K, Michels N, de Vriendt T, Vyncke K, et al Intercorrelations between serum, salivary, and hair cortisol and child-reported estimates of stress in elementary school girls. Psychophysiology. 2012; 49(8):1072–81. 10.1111/j.1469-8986.2012.01396.x 22725679

[35] Gerber M, Endes K, Brand S, Herrmann C, Colledge F, Donath L. In 6- to 8-year-old children, hair cortisol is associated with body mass index and somatic complaints, but not with stress, health-related quality of life, blood pressure, retinal vessel diameters, and cardiorespiratory fitness. Psychoneuroendocrinology, 2017; 76:1–10. 10.1016/j.psyneuen.2016.11.008 27865992

[36] Mayer SE, Lopez-Duran NL, Sen S, Abelson JL. Chronic stress, hair cortisol and depression: A prospective and longitudinal study of medical internship. Psychoneuroendocrinology. 2018; 92:57–65. 10.1016/j.psyneuen.2018.03.020 29627713 PMC5924646

[37] Russell E, Koren G, Rieder M, Van Uum S. Hair cortisol as a biological marker of chronic stress: current status, future directions and unanswered questions. Psychoneuroendocrinology. 2012; 37(5):589–601. 10.1016/j.psyneuen.2011.09.009 21974976

[38] Slominski R, Rovnaghi CR, Anand KJ. Methodological Considerations for Hair Cortisol Measurements in Children. Ther Drug Monit. 2015 37(6):812–20. 10.1097/FTD.0000000000000209 25811341 PMC4581896

[39] Oldehinkel AJ, Ormel J, Bosch NM, Bouma E, Van Roon AM, Rosmalen JG. Stressed out? Associations between perceived and physiological stress responses in adolescents: The TRAILS study. Psychophysiology. 2011; 48:441–52. 10.1111/j.1469-8986.2010.01118.x 21361964

[40] Adam EK. Transactions among adolescent trait and state emotion and diurnal and momentary cortisol activity in naturalistic settings. Psychoneuroendocrinology. 2006; 31:664–79. 10.1016/j.psyneuen.2006.01.010 16584847

[41] Kalra S, Einarson A, Karaskov T, van Uum S, Koren G. The relationship between stress and hair cortisol in healthy pregnant women. Clin Invest Med. 2007; 30(2): E103–7. 17716540 10.25011/cim.v30i2.986

[42] Rietschel L, Streit F, Zhu G, McAloney K, Kirschbaum C, Frank J, et al Hair cortisol and its association with psychological risk factors for psychiatric disorders: A pilot study in adolescent twins. Twin Res Hum Genet. 2016; 19:438–46. 10.1017/thg.2016.50 27374135

[43] Van Uum S, Sauve B, Fraser L-A, Morley-Forster P, Paul TL, Koren G. Elevated content of cortisol in hair of patients with severe chronic pain: a novel biomarker for stress. Stress. 2008; 11(6):483–8. 10.1080/10253890801887388 18609301

[44] Skoluda N, Dettenborn L, Stalder T, Kirschbaum C. Elevated hair cortisol concentrations in endurance athletes. Psychoneuroendocrinology. 2012; 37(5):611–7. 10.1016/j.psyneuen.2011.09.001 21944954

[45] Gidlow CJ, Randall J, Gillman J, Silk S, Jones MV. Hair cortisol and self-reported stress in healthy, working adults. Psychoneuroendocrinology. 2016; 63:163–9. 10.1016/j.psyneuen.2015.09.022 26447679

[46] Stalder T, Steudte-Schmiedgen S, Alexander N, Klucken T, Vater A, Wichmann S, et al Stress-related and basic determinants of hair cortisol in humans: A meta-analysis. Psychoneuroendocrinology. 2017; 77:261–74. 10.1016/j.psyneuen.2016.12.017 28135674

[47] Maldonado EF, Fernandez FJ, Trianes MV, Wesnes K, Petrini O, Zangara A. Cognitive performance and morning levels of salivary cortisol and [alpha]-amylase in children reporting high vs. low daily stress perception. Span J Psychol. 2008; 11(1):3–15. 18630643 10.1017/s1138741600004066

[48] Fuchs A, Jaite C, Neukel C, Dittrich K, Bertsch K, Kluczniok D, et al Link between children’s hair cortisol and psychopathology or quality of life moderated by childhood adversity risk. Psychoneuroendocrinology. 2018; 90:52–60. 10.1016/j.psyneuen.2018.02.003 29433073

[49] Gray NA, Dhana A, Van Der Vyver L, Van Wyk J, Khumalo NP, Stein DJ. Determinants of hair cortisol concentration in children: A systematic review. Psychoneuroendocrinology. 2018; 87:204–14. 10.1016/j.psyneuen.2017.10.022 29112905

[50] Kamps AW, Molenmaker M, Kemperman R, van der Veen BS, Bocca G, Veeger NJ. Children with asthma have significantly lower long-term cortisol levels in their scalp hair than healthy children. Acta Paediatr. 2014; 103(9):957–61. 10.1111/apa.12685 24814069

[51] Föcker M, Stalder T, Kirschbaum C, Albrecht M, Adams F, de Zwaan M, et al Hair cortisol concentrations in adolescent girls with anorexia nervosa are lower compared to healthy and psychiatric controls. Eur Eat Disord Rev. 2016; 24: 531–5. 10.1002/erv.2466 27509907

[52] Noppe G, van Rossum EF, Vliegenthart J, Koper JW, van den Akker EL. Elevated hair cortisol concentrations in children with adrenal insufficiency on hydrocortisone replacement therapy. Clin Endocrinol. 2014; 81(6):820–5. 10.1111/cen.12551 25039686

[53] Michels N, Van de Wiele T, de Henauw S. Chronic psychosocial stress and gut health in children: associations with calprotectin and fecal short-Chain fatty acids. Psychosom Med. 2016; 79(8):927–35. 10.1097/PSY.0000000000000413 27787408

[54] Olstad DL, Ball K, Wright C, Abbott G, Brown E, Turner AI. Hair cortisol levels, perceived stress and body mass index in women and children living in socioeconomically disadvantaged neighborhoods: the READI study. Stress, 2016; 19(2):158–67. 10.3109/10253890.2016.1160282 27023344

[55] White LO, Ising M, von Klitzing K, Sierau S, Michel A, Klein AM, et al Reduced hair cortisol after maltreatment mediates externalizing symptoms in middle childhood and adolescence. J Child Psychol Psyc. 2017; 58(9):998–1007. 10.1111/jcpp.12700 28244601 PMC5570647

[56] Zhang Q, Chen Z, Chen S, Xu Y, Deng H. Intraindividual stability of cortisol and cortisone and the ratio of cortisol to cortisone in saliva, urine and hair. Steroids. 2017; 118:61–7. 10.1016/j.steroids.2016.12.008 27998757

[57] Daughters SB, Gorka SM, Matusiewicz A, Anderson K. Gender Specific Effect of Psychological Stress and Cortisol Reactivity on Adolescent Risk Taking. J Abnorm Child Psychol. 2013; 41:749–58. 10.1007/s10802-013-9713-4 23338478 PMC3681843

[58] Lu Q, Pan F, Ren L, Xiao J, Tao F. Sex differences in the association between internalizing symptoms and hair cortisol level among 10–12 year-old adolescents in China. PLoS One. 2018; 13(3): e0192901 10.1371/journal.pone.0192901 29522544 PMC5844552

[59] Moksnes UK, Moljord IE, Espnes GA, Byrne DG. The association between stress and emotional states in adolescents. The role of gender and self-esteem. Pers Individ Differ. 2010; 49:430–5. 10.1016/j.paid.2010.04.012

[60] Rippe RC, Noppe G, Windhorst DA, Tiemeier H, van Rossum EF, Jaddoe VW, et al Splitting hair for cortisol? Associations of socio-economic status, ethnicity, hair color, gender and other child characteristics with hair cortisol and cortisone. Psychoneuroendocrinology. 2016; 66:56–64. 10.1016/j.psyneuen.2015.12.016 26773401

[61] Groeneveld MG, Vermeer HJ, Linting M, Noppe G, van Rossum EFC, van IJzendoorn MH. Children’s hair cortisol as a biomarker of stress at school entry. Stress. 2013; 16(6):711–5. 10.3109/10253890.2013.817553 23786528

[62] Veldhorst M. A., Noppe G., Jongejan M. H., Kok C. B., Mekic S., Koper J. W., et al Increased scalp hair cortisol concentrations in obese children. J Clin Endocr Metab. 2014; 99(1):285–90. 10.1210/jc.2013-2924 24384019

[63] Boeckel MG, Viola TW, Daruy-Filho L, Martinez M, Grassi-Oliveira R. Intimate partner violence is associated with increased maternal hair cortisol in mother–child dyads. Compr Psychiatry. 2017; 72:18–24. 10.1016/j.comppsych.2016.09.006 27693887

[64] Papafotiou C, Christaki E, van den Akker EL, Wester VL, Apostolakou F, Papassotiriou I, et al Hair cortisol concentrations exhibit a positive association with salivary cortisol profiles and are increased in obese prepubertal girls. Stress, 2017; 20(2):217–22. 10.1080/10253890.2017.1303830 28270027

[65] Simmons JG, Badcock PB, Whittle SL, Byrne ML, Mundy L, Patton GC, et al The lifetime experience of traumatic events is associated with hair cortisol concentrations in community-based children. Psychoneuroendocrinology. 2016; 63:276–81. 10.1016/j.psyneuen.2015.10.004 26529051

[66] Ursache A, Merz EC, Melvin S, Meyer J, Noble KG. Socioeconomic status: hair cortisol and internalizing symptoms in parents and children. Psychoneuroendocrinology. 2017; 78:142–50. 10.1016/j.psyneuen.2017.01.020 28199857 PMC5421817

[67] Marshall WA, Tanner JM. Variations in pattern of pubertal changes in girls. Arch Dis Child. 1969; 44(235): 291–303. 5785179 10.1136/adc.44.235.291 PMC2020314

[68] Marshall WA, Tanner JM. Variations in the pattern of pubertal changes in boys. Arch Dis Child. 1970; 45(239): 13–23. 5440182 10.1136/adc.45.239.13 PMC2020414

[69] Cohen S, Kamarck T, Mermelstein R. A global measure of perceived stress. J Health Soc Behav. 1983; 24(4):385–96. 6668417

[70] Herrero J, Meneses J. Short Web-based versions of the perceived stress (PSS) and Center for Epidemiological Studies-Depression (CESD) Scales: A comparison to pencil and paper responses among Internet users. Comput Hum Behav. 2006; 22:830–46. 10.1016/j.chb.2004.03.007

[71] Cohen S, Williamson G. Perceived stress in a probability sample of the US In: Spacapam S, Oskamp S, editors. The social psychology of health: Claremont symposium on applied social psychology. Newbury Park, CA: Sage; 1988 p. 31–67.

[72] Thomson S, Koren G, Fraser LA, Rieder M, Friedman TC, Van Uum SH. Hair analysis provides a historical record of cortisol levels in Cushing’s syndrome. Exp Clin Endocrinol Diabet. 2010; 118(2):133–8. 10.1055/s-0029-1220771 19609841 PMC2945912

[73] D’Este P, Amara N, Olmos-Peñuela J. Fostering novelty while reducing failure: Balancing the twin challenges of product innovation. Technol Forecast Soc Change, 2016; 113: 280–92. 10.1016/j.techfore.2015.08.011

[74] Trueba AF, Smith NB, Auchus RJ, Ritz T. Academic exam stress and depressive mood are associated with reductions in exhaled nitric oxide in healthy individuals. Biol Psychol. 2013; 93(1): 206–12. 10.1016/j.biopsycho.2013.01.017 23410759

[75] Lazarus SR, Folkman S. Stress, appraisal and coping. New York: Springer; 1984.

